# Longitudinal Course of Myotonic Dystrophy Type 1 With Gait Training Using a Hybrid Assistive Limb: A Case Report

**DOI:** 10.7759/cureus.71030

**Published:** 2024-10-07

**Authors:** Ryo Nakazawa, Kazunori Koseki, Kenichi Yoshikawa, Akira Matsushita, Yutaka Kohno

**Affiliations:** 1 Department of Physical Therapy, Ibaraki Prefectural University of Health Sciences Hospital, Ami, JPN; 2 Department of Neurosurgery, Ibaraki Prefectural University of Health Sciences Hospital, Ami, JPN; 3 Department of Neurology, Ibaraki Prefectural University of Health Sciences Hospital, Ami, JPN; 4 Center for Medical Sciences, Ibaraki Prefectural University of Health Sciences, Ami, JPN

**Keywords:** case report, gait training, hybrid assistive limb, long-term observation, myotonic dystrophy type 1

## Abstract

Muscle dystrophy type 1 is the most common form of muscular dystrophy and is characterized by progressive distal dominant muscle weakness, muscle atrophy, and myotonic phenomena. Patients with progressive neuromuscular diseases such as myotonic dystrophy type 1 are prone to muscle weakness, movement disorders, and fatigue due to their underlying disease, which may limit their physical activity and ambulation.

Six courses of gait training with a hybrid assistive limb (HAL) were performed in an adult male with myotonic dystrophy type 1 over 4.5 years. The two-minute walking distance and knee joint strength tended to increase, and the Timed Up and Go test time tended to decrease with gait training with HAL before and after each intervention. In the long term, the two-minute walking distance decreased slowly, but the Timed Up and Go and knee joint strength tended to be maintained. Functional ambulation category and activities of daily living levels were also maintained. These results suggest that intermittent gait training may be an effective rehabilitation method for maintaining gait independence and activities of daily living in patients with myotonic dystrophy type 1. The frequency and number of interventions should be considered for further effectiveness.

## Introduction

Myotonic dystrophy type 1 is the most common form of muscular dystrophy and is characterized by progressive distal dominant muscle weakness, muscle atrophy, and myotonic phenomena. The average life expectancy is reported to be 48-60 years, and approximately half of the patients are partially or completely wheelchair-bound shortly before death [1,2]. It is widely accepted that rehabilitation interventions, including exercise therapy, are an effective means of maintaining activities of daily living. Furthermore, there is an increasing body of evidence to suggest that moderate-intensity aerobic exercise, or a combination of aerobic and muscle-strengthening exercise, is beneficial for patients with muscle disease without causing harmful muscle damage [3]. Nevertheless, a definitive and efficacious exercise regimen for individuals diagnosed with myotonic dystrophy type 1 remains to be established. We believe that it is important to identify rehabilitation methods that can maintain and improve the walking ability of patients with myotonic dystrophy type 1.

Recently, gait training using robotics has been implemented for neuromuscular diseases [4]. Gait training using hybrid assistive limb (HAL) showed significant improvements in gait function after one course of nine interventions in the first randomized controlled trial for neuromuscular diseases [5]. However, the effect of intermittent long-term HAL training on myotonic dystrophy type 1 remains unknown.

In this case report, a patient with myotonic dystrophy type 1 underwent six courses of training with HAL over a period of approximately 4.5 years, and the patient's gait endurance, dynamic balance ability, and muscle strength were maintained over a long period of time.

## Case presentation

The patient was a male with myotonic dystrophy type 1, in his 40s, 168 cm tall, and 76.8 kg at the time of initial admission. He had been aware of his upper limb disability since his teenage years but was able to lead a social life and work in warehouse management. In his 30s, he was diagnosed with myotonic dystrophy type 1 by genetic testing. At the beginning of his 40s, he resigned from his job because of difficulty in continuing his work and was admitted to the HAL program (details are described in the section below) for the first time. The patient had a history of bilateral cataract surgery, without other significant complications. The patient's physical condition at the time of initial admission was as follows: The manual muscle test of the upper and lower extremities revealed four levels of proximal muscles (flexion of shoulder and elbow joints, hip joint flexion and extension, knee joint extension) and two levels of distal muscles (dorsiflexion of wrist and ankle joints), with distal muscle weakness (grip strength was 2 kg on the right and 1 kg on the left), percussive myotonia, dysarthria, and dysphagia, and the need to use a thickener when drinking [6]. He could perform basic activities such as turning over, getting up, and standing up. Indoor/outdoor ambulation is possible; however, in winter, the patient requires a cane because of reduced mobility. He could walk 100-200 m at a time, and during hospitalization, he needed to use a walking wheelchair or wheelchair depending on his fatigue. His functional independence measure (FIM) motor score was 87, and his activities of daily living were at an independent level; however, he sometimes needed assistance with changing clothes in cold weather [7]. In his home life, he exercised to the extent of walking once every two days (approximately 1,000 steps). There has been no significant change in these 4.5 years with respect to his exercise habits.

HAL program

One HAL program was conducted in an inpatient setting for approximately one month. The HAL program is a training program combining training with HAL and a conventional rehabilitation program. Conventional rehabilitation consists of physical therapy, occupational therapy, and speech therapy, with each therapy lasting 40-60 min per day. The program included stretching, strength training, balance practice, and activities of daily living practice (dressing, toileting, bathing, and others) while considering the fatigue status of the HAL training.

HAL for medical use (both lower limb types, model number ML-05, small size, Cyberdyne Corporation, Tsukuba, Japan) was used to provide HAL training to patients and was carried out according to the HAL package insert and proper usage guide. There are two modes of motion assistance for the hip and knee joints in the HAL [8]. The Cybernic Voluntary Control (CVC) mode provides physical support and action according to the operator’s voluntary intention caused by bioelectrical signals, including muscle activity. Cybernic Autonomous Control (CAC) autonomously provides the desired functional motion generated according to the wearer’s body constitution, conditions, and purposes of motion support. We used the CVC mode for the motion assistance of each joint. The torque tuner (power of joint movement assistance), balance tuner (balancing hip and knee flexion and extension), and walking mode (walking speed) were adjusted appropriately by the therapist so that the patient could walk comfortably on level ground. In this case, HAL training was conducted up to nine times during each course, two to three times a week, and the actual gait training time was less than 20 min (excluding rest time). This was performed by considering the patient's fatigue level. A walker (All-in-One Walking Trainer; Healthcare Lifting Specialist, Ropox A/S, Denmark) with a harness was used as a fall prevention measure during HAL training. HAL programs were performed six times on the patient in his 40s. The patient was hospitalized six times approximately every other year, with HAL training being provided during hospitalization (Table 1).

**Table 1:** Details of each intervention period and HAL setting *Pre-intervention FAC, functional ambulation categories; FIM, functional independence measure; AM, assist mode

	Age*	FAC*	FIM-motor*	Intervention duration	Total HAL training sessions	Hip joint AM（R/L）	Knee joint AM（R/L）
Course 1	42	4	87	2018/9/11 - 10/2	9	CVC/CVC	CVC/CVC
Course 2	43	4	87	2019/1/29 - 2/19	9	CVC/CVC	CVC/CVC
Course 3	44	4	87	2020/3/3 - 3/24	9	CVC/CVC	CVC/CVC
Course 4	45	4	86	2021/2/17 - 3/8	9	CVC/CVC	CVC/CVC
Course 5	46	4	85	2022/1/27 - 2/16	9	CVC/CVC	CVC/CVC
Course 6	47	4	83	2023/2/21 - 3/13	9	CVC/CVC	CVC/CVC

Outcome measures

The measurements were performed before and after each HAL program. We examined the two-minute walking distance using the two-minute walking test as an index of walking durability, the Timed Up and Go test as an index of dynamic balance ability, and bilateral knee joint flexion/extension torque as an index of lower limb muscle strength. Creatine kinase (CK) levels were measured as an indicator of muscle damage.

The two-minute walking test was performed along a walking path with a circumference of 45 m. The patient was instructed to walk as long as possible for 2 min, and the walking distance was measured. The six-minute walking test is often employed as a functional assessment for neuromuscular diseases and has been shown to be useful [9]. In neuromuscular diseases, the two-minute walking test correlates strongly with and can be substituted for the six-minute walking test [10]. In the Timed Up and Go test, the walking speed was set to a comfortable speed, and the rotation direction was chosen by the participants so that they could easily turn. The Timed Up and Go test has been reported to be useful for diagnosing myotonic dystrophy type 1 [11]. Knee joint flexion/extension torque was measured using a manual muscular strength meter (manual muscular strength meter Moby, SAKAI Medical Co., Ltd., Japan) in the sitting position. Measurements were performed with a belt fixed to the patient’s shank and the objects [12]. The maximum knee joint extension and flexion torques were measured in two sets, and the average values of the two trials were adopted. To normalize the measurements, the product of the measured torque (N) and distance (m) from the center of the knee joint to the measurement equipment was divided by the patient's weight (kg). The left and right values were added to obtain extension and flexion torque values. After each HAL program period, the participants were asked to describe their impressions of the HAL training in an open-ended form.

Treatment progress

**Figure 1:**
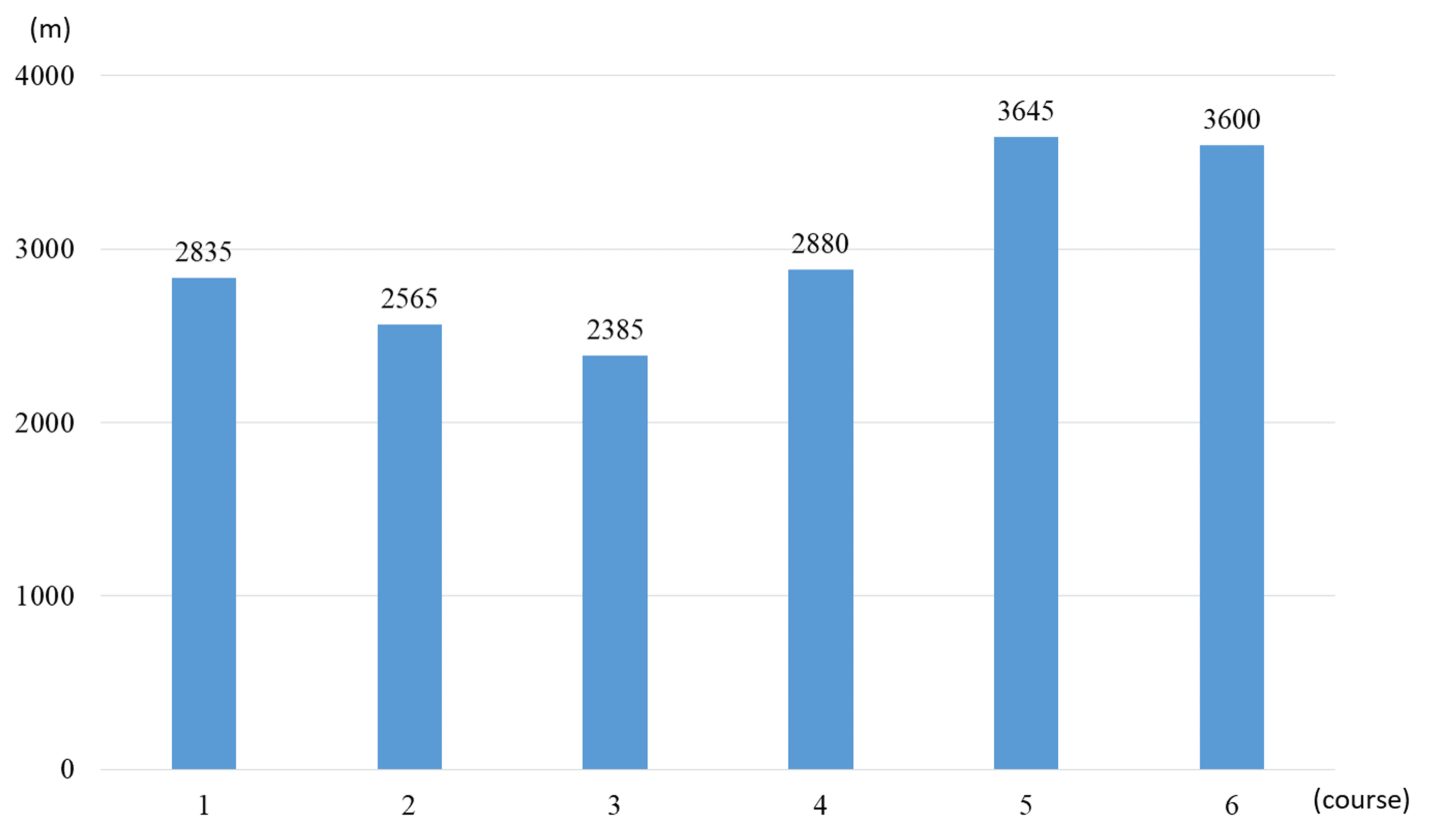
Total walking distance for each course The vertical axis represents distance and the horizontal axis represents course.

The patient completed six HAL training within a 4.5-year period; no adverse events such as falls or excessive fatigue occurred during the HAL programs. No adverse events (abrasions, significant fatigue, or pain) occurred during HAL training that would have caused the patient to fall or stop the training. The patient was able to complete the HAL program with no worsening of CK levels, except for an increase before and after the fifth and sixth courses. The total walking distance for each course is shown in Figure 1. The patient’s walking independence (functional ambulation category (FAC)) was maintained without decline for approximately 4.5 years and activities of daily living (FIM motor score) decreased by only three points.

**Figure 2:**
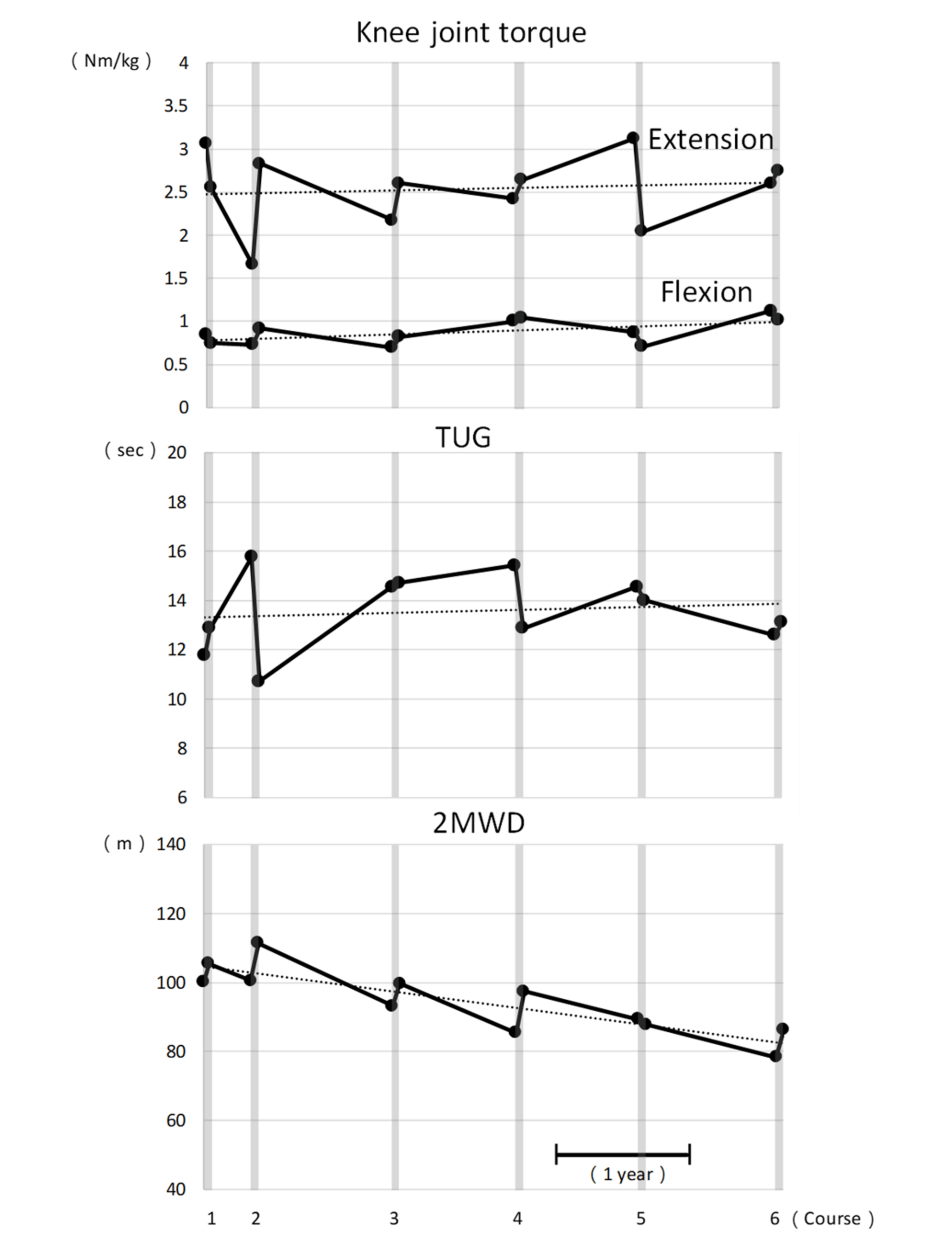
Change in each evaluation value Dotted lines indicate approximate straight lines for each data set. The lower right bar is the scale of one year. The horizontal axis is the time axis. Vertical lines indicate each measurement.

The measurements of the two-minute walking distance, Timed Up and Go test, CK, maximum knee joint extension and flexion torque, FAC, and FIM motor are shown in Table 2. The knee joint maximal extension and flexion torques, the two-minute walking distance, and the Timed Up and Go test results are shown in Figure 2. The two-minute walking distance increased before and after HAL training in all treatment periods except for the fifth. Timed Up and Go test times increased slightly during the first, third, and sixth treatment periods but shortened during the rest period. Left and right knee extensor strength decreased during the first and fifth treatment periods but increased otherwise. Left and right knee flexor strength decreased during the first, fifth, and sixth treatment periods but increased during the rest of the treatments. The two-minute walking distance tended to decrease, and the Timed Up and Go test and lower limb muscle strength tended to be maintained over the long-term course of the study. The questionnaire to the subject was as follows: "I did not feel any physical pain during HAL training"; "My legs felt lighter after walking with HAL."

**Table 2:** Outcome measures in each period 2MD: walking distance measured by two-minute walking test; TUG, timed up and go test; CK, creatine kinase; KE, knee extensor strength (right and left); KF, knee flexor strength (right and left); FAC: functional ambulation categories; FIM, functional independence measure

	Pre1	Post1	Pre2	Post2	Pre3	Post3	Pre4	Post4	Pre5	Post5	Pre6	Post6
2MWD (m)	100.25	105.45	100.55	111.35	93.15	99.65	85.45	97.45	89.35	87.85	78.35	86.35
TUG (sec)	11.78	12.88	15.75	10.72	14.56	14.71	15.43	12.88	14.56	14.00	12.59	13.11
CK (U/L)	368	323	318	260	275	235	322	266	314	370	263	370
KE (Nm/Kg)	3.06	2.55	1.66	2.83	2.17	2.60	2.42	2.64	3.12	2.04	2.60	2.75
KF (Nm/Kg)	0.85	0.75	0.73	0.92	0.70	0.82	1.00	1.04	0.87	0.71	1.12	1.02
FAC	4	4	4	4	4	4	4	4	4	4	4	4
FIM motor	87	87	87	87	87	87	86	86	85	86	83	84

## Discussion

Patients with progressive neuromuscular diseases such as myotonic dystrophy type 1 are prone to muscle weakness, movement difficulties, and fatigue due to their underlying disease, which may limit their physical activity and ambulation [13]. However, this patient was able to maintain walking independence (FAM) and activities of daily living (FIM motor) for approximately 4.5 years, with long-term intermittent HAL training.

In the case reported here, the patient was able to continue HAL training for six courses over 4.5 years. In all courses, CK values remained between 235 and 370 U/L, and the procedures were performed safely without falls or other adverse events.

Of the six courses of HAL training, the two-minute walking distance showed improvement in five. Similarly, the Timed Up and Go test improved in three courses, maximum knee joint extension torque in four courses, and flexion torque in three courses. Gait endurance, dynamic balance capacity, and lower-extremity muscle strength often increase in the short term. These results were consistent with those of previous studies. The fifth course involved a greater degree of walking than the preceding four, and the patient may have exhibited signs of fatigue during the post-HAL assessment. This was confirmed by the patient himself. It is possible that this may have had an impact on the results of the measurement of lower extremity muscle strength with maximal effort. Similarly, it is possible that this may have affected the results of the two-minute walking distance, which assesses endurance.

Over the 4.5-year period, the patient's knee extensor and flexor strength and dynamic balance ability were maintained, with a very gradual decline in walking endurance. A prospective cohort study of patients with myotonic dystrophy type 1 over a five-year period reported significant decreases in knee extension and flexion muscle strength and a significant increase in the Timed Up and Go test time required over five years [14]. They reported that both decreases were more pronounced in the male group. This patient’s knee joint strength and dynamic balance ability were generally maintained, and we believe that regular HAL training maintains physical function. This patient was treated approximately every year, but a previous case report showed an increase in the two-minute walking distance with HAL training intervention at six-month intervals [15]. In order to maintain walking endurance with HAL training, we infer that it is necessary to examine the frequency of intervention, such as implementing an intervention at least once every six months, and to reexamine the exercise load, such as increasing the amount of exercise per course.

In addition, the tolerability and safety of long-term interventions were discussed. By adjusting the amount of joint assistance, the HAL enables the patient to practice walking with an optimum amount of load according to the patient's physical condition, and the hoist can be used to ensure a safe walking volume. A comparison of the CK values before and after HAL training showed that they did not exceed approximately 1.5 times the upper normal limit before and after each course, suggesting that there was little muscle loading from the exercise. We believe that long-term intermittent HAL training is effective and safe for patients with myotonic dystrophy type 1 and maintains and improves gait endurance and lower-extremity muscle strength. In addition to securing the amount of walking, HAL training allows the patient to experience walking movements (e.g., the center of gravity shift and postural control during a change of direction) that cannot be experienced on a treadmill, which is common in other robot-assisted gait training, by practicing walking on the ground. Hence, we believe that the time required for the Timed Up and Go test was reduced, and dynamic balance ability was maintained.

## Conclusions

In this patient, continuous HAL training improved walking endurance, dynamic balance ability, and muscle strength in the short term. Long-term intermittent HAL training maintains knee extension, flexion muscle strength, and dynamic balance. Gait endurance declined slowly, suggesting that gait independence and activities of daily living capacity were maintained. It is essential to consider the frequency of intervention and the amount of exercise in order to maintain and improve the patient's physical function.

## References

[1] Turner C, Hilton-Jones D (2010). The myotonic dystrophies: diagnosis and management. J Neurol Neurosurg Psychiatry.

[2] de Die-Smulders CE, Höweler CJ, Thijs C (1998). Age and causes of death in adult-onset myotonic dystrophy. Brain.

[3] Cup EH, Pieterse AJ, Ten Broek-Pastoor JM (2007). Exercise therapy and other types of physical therapy for patients with neuromuscular diseases: a systematic review. Arch Phys Med Rehabil.

[4] Rodríguez-Fernández A, Lobo-Prat J, Font-Llagunes JM (2021). Systematic review on wearable lower-limb exoskeletons for gait training in neuromuscular impairments. J Neuroeng Rehabil.

[5] Nakajima T, Sankai Y, Takata S (2021). Cybernic treatment with wearable cyborg Hybrid Assistive Limb (HAL) improves ambulatory function in patients with slowly progressive rare neuromuscular diseases: a multicentre, randomised, controlled crossover trial for efficacy and safety (NCY-3001). Orphanet J Rare Dis.

[6] Cuthbert SC, Goodheart GJ Jr (2007). On the reliability and validity of manual muscle testing: a literature review. Chiropr Osteopat.

[7] Ottenbacher KJ, Hsu Y, Granger CV, Fiedler RC (1996). The reliability of the functional independence measure: a quantitative review. Arch Phys Med Rehabil.

[8] Sankai Y (2010). HAL: Hybrid Assistive Limb Based on Cybernics. Robotics Research. Springer Tracts in Advanced Robotics.

[9] Kierkegaard M, Tollbäck A (2007). Reliability and feasibility of the six minute walk test in subjects with myotonic dystrophy. Neuromuscul Disord.

[10] Witherspoon JW, Vasavada R, Logaraj RH (2019). Two-minute versus 6-minute walk distances during 6-minute walk test in neuromuscular disease: Is the 2-minute walk test an effective alternative to a 6-minute walk test?. Eur J Paediatr Neurol.

[11] Hammarén E, Ohlsson JA, Lindberg C (2012). Reliability of static and dynamic balance tests in subjects with myotonic dystrophy type 1. Adv Physiother.

[12] Katoh M, Yamasaki H (2009). Comparison of reliability of isometric leg muscle strength measurements made using a hand-held dynamometer with and without a restraining belt. J Phys Ther Sci.

[13] Mcdonald CM, Mcdonald M (2002). Physical activity, health impairments, and disability in neuromuscular disease literature review adaptations to disuse and exercise. Am J Phys Med Rehabil.

[14] Hammarén E, Kjellby-Wendt G, Lindberg C (2015). Muscle force, balance and falls in muscular impaired individuals with myotonic dystrophy type 1: a five-year prospective cohort study. Neuromuscul Disord.

[15] Nakatsu D, Matsui M, Yonenobu Y, Toyooka K, Inoue K, Saito T (2021). A case study of a patient with myotonic dystrophy type 1 whose gait disturbance was improved by gait training with hybrid assistive limbs (Japanese). Rinsho Shinkeigaku.

